# A Case of Iritis Serosa

**Published:** 1881-02

**Authors:** E. J. Gardiner

**Affiliations:** Assistant Surgeon for the Illinois Charitable Eye and Ear Infirmary


### ARTICLE IV.

### A CASE OF IRITIS SEROSA. By Dr. E. J. Gardiner, Assistant Surgeon for the Illinois Charitable Eye and Ear Infirmary.

Plastic iritis is so well characterized by its severe manifestations that the diagnosis is easy for any physician who has attended an ophthalmic clinic. The symptoms of idiopathic or plastic iritis are: pericorneal congestion, contraction and sluggishness of the pupil, posterior synechia, which makes the pupil irregular, discoloration of the iris, lachrymation and severe pain about the supraorbital region, sometimes extending to all the sensitive branches of the fifth nerve. If the disease is in its first stage, when the pupil is not yet contracted, the pericorneal injection not marked, and the pain not felt, some little difficulty may be found in making the diagnosis; this, however, is the exception, not the rule.

In iritis serosa the condition varies entirely. It is by nature an insidious disease, and may run its course, implicating both the ciliary body and the choroid, with such small manifestations, that even the most experienced may be duped by the disease. Of course cases differ very much, and while one patient may present himself with an eye, the appearances of which would not lead one to suspect any trouble of the iris, another will present himself with a collection of symptoms, which will easily lead to the correct diagnosis.

Iritis serosa and hydromeningitis, are names given to a special form of iritis, characterized by a peculiar implication of the posterior layer of the cornea (Descemet's membrane), which becomes covered with little grayish spots, so loosely attached that a drop of water, allowed to flow over the membrane, will wash them away. These little spots sometimes group into a circle, but more frequently into a triangular form, the basis being directed toward the periphery of the cornea. There exists generally, a hypersecretion of aqueous humor, which not only increases the tension, but secondarily produces a slight dilatation of the pupil. The discoloration of the iris is not always found, and, although the pericorneal injection may be counted as a pathognomonic symptom, it is not always present. The plastic exudations forming synechia, so common in idiopathic iritis, are never found in serous iritis. The characteristic symptom of this disease is the punctured appearance of the posterior surface of the cornea. The little dots can be seen with the ophthalmoscope used for the upright image, but by focal illumination they can be better examined and located. This form of iritis is generally very refractive for treatment, and relapses are very frequent. During my assistantship to Dr. H. Knapp, of New York, I had occasion to examine several cases of this disease, among them, one of the insidious form; without this experience, I think that I would have been deceived by the following case:

Mr. M. P., age twenty-four, of strong constitution, neither smokes nor drinks, states that, about a week ago, he experienced a slight pain about his right supraorbital region, after which he noticed that the sight was blurred in his right eye. No change for the better having taken place during the week, he concluded to consult an oculist. *Stat. pres:* Conjunctiva slightly hyperæmic, *no pericorneal injection;* cornea clear, iris somewhat discolored, (patient states that it had always been of that color); pupil normal, reacting well to light. While making the ophthalmoscopic examination in the upright image, I noticed a peculiar dotted appearance of the cornea. With the focal illumination I found that these dots were situated on Descemet's membrane, and grouped in a triangular form, the basis directed toward the periphery of the cornea. They were so small however, that I was obliged to center all my attention to discover those situated at the vertex of the triangle. I found the fundus normal; tension was also normal; vision $\frac{20}{100}$; left eye normal, $V = \frac{20}{20}$. The patient stated that he could not bear a bright light with his right eye, and that *sometimes* toward the evening he experienced an uneasy sensation, with *a little* pain over the eyebrow. My mind was made up. The case before me was one of incipient iritis serosa. I instilled a couple of drops of a four-gr. solution of atropia, and was not much surprised to find the pupil very slow in dilating. After repeating the instillations three times the desired effect was obtained. Prescribed atrop. sulph. gr. ij to the ounce of water; calomel, gr. one sixth; three times a day internally. Two days afterward, I saw Mr. P., and found that the pupils had not entirely dilated. The dots on Descemet's membrane were more abundant and larger. No pain had been experienced since the first instillation of atropia. Calomel to be continued, and the atropia to be used, one drop every two hours until the pupil should be fully dilated, then one drop three times a day. Two days afterward, the pupil was fully dilated. From this time improvement continued very rapidly; so much so, that afer six weeks' treatment, I considered the eye cured. The condition as noticed on my record book was, "No pericorneal injection, pupil fully dilated, no pain, very slight haziness of cornea; $V = \frac{20}{40}$." On account of the haziness and a few hardly perceptible dots on the cornea, I advised Mr. P. to continue using his two-gr. solution of atropia, and to still use a protective bandage, but to discontinue the iodide of potash, which he had been taking for about two weeks. I saw no more of him until about two weeks afterward, when he presented himself very much alarmed on account of the severe symptoms which had manifested themselves in his eye. He stated that improvement had continued since I last saw him, until the night previous, when he was seized with severe pain in the eyeball and brow. The eye now presented a well-marked pericorneal injection. The cornea was hazy; the dots on Descemet's membrane were numerous and large. The details of the fundus could not be made out on account of the haziness of the cornea, the tension, which, during the previous attack had remained normal, had now increased to + 91. I instilled a one per cent. solution of eserine, prescribed a mild cathartic, protective bandage and absolute rest in bed. On the morrow I saw the patient; the pain had entirely subsided, tension had decreased to the normal state, pupil contracted, pericorneal injection well-marked yet. Atropia and warm fomentations, combined with proper hygiene soon brought the eye to its normal condition without any further relapses. The last time I saw the eye the cornea was perfectly clear, pupil normal, retaining the same color, however, that it presented when I first examined the case. No appreciable changes had taken place in the fundus. Vision had improved to about $\frac{20}{20}$. I have lately seen Mr. P.; he informed me that his eye had given him no further trouble.

This case illustrates very well the insidious form of iritis serosa; its long duration and sudden relapses. I have had occasion to treat several similar cases, but in none that I can remember, did I meet with such a lack of iritic symptoms.

