# Errata

**Published:** 1881-03

**Authors:** 


ERRATA.—The article entitled "A case of Iritis Serosa," published in the February number of the Journal went to press, the proof not being corrected. The following are misprints:

Page 133, line 2, for should read to; page 133, line 6, congestion should read injection; page 133, line 7, synechia should read synechiæ; page 133, line 7, makes should read make; page 134, line 3, appearances should read appearance; page 134, line 7, and should read or; page 134, line 20, synechia should read synechiæ; page 134, line 22, punctured should read punctated; page 134, line 25, for should read to; page 135, line 28, noticed should read noted; page 136, line 7, T + 91 should read T + 1.

THE Illinois State Board of Health is now engaged in preparing the second edition of the "Register of Physicians and Midwives," and will be obliged to the profession if they will call attention to mistakes and changes of location or omissions. Secretaries of medical societies are also requested to send the roster of their officers, and the names, age and cause of death of any medical men who have died within the last year.

The Board is anxious to make the Register as perfect as possible. It is also important to every one that his or her record is correct.

